# Sequencing the Plastid Genome of Giant Ragweed (*Ambrosia trifida*, Asteraceae) From a Herbarium Specimen

**DOI:** 10.3389/fpls.2019.00218

**Published:** 2019-02-28

**Authors:** Gaurav Sablok, Ali Amiryousefi, Xiaolan He, Jaakko Hyvönen, Péter Poczai

**Affiliations:** ^1^Finnish Museum of Natural History (Botany Unit), University of Helsinki, Helsinki, Finland; ^2^Organismal Evolution and Biology, Faculty of Biology and Environmental Sciences, Viikki Plant Science Centre, University of Helsinki, Helsinki, Finland

**Keywords:** *Ambrosia trifida*, chloroplast genome, genome skimming, glyphosate, herbicide resistance, invasive plants, noxious weeds, ragweed

## Abstract

We report the first plastome sequence of giant ragweed (*Ambrosia trifida*); with this new genome information, we assessed the phylogeny of Asteraceae and the transcriptional profiling against glyphosate resistance in giant ragweed. Assembly and genic features show a normal angiosperm quadripartite plastome structure with no signatures of deviation in gene directionality. Comparative analysis revealed large inversions across the plastome of giant ragweed and the previously sequenced members of the plant family. Asteraceae plastid genomes contain two inversions of 22.8 and 3.3 kb; the former is located between *trn*S-GCU and *trn*G-UCC genes, and the latter between *trn*E-UUC and *trn*T-GGU genes. The plastid genome sequences of *A. trifida* and the related species, *Ambrosia artemisiifolia*, are identical in gene content and arrangement, but they differ in length. The phylogeny is well-resolved and congruent with previous hypotheses about the phylogenetic relationship of Asteraceae. Transcriptomic analysis revealed divergence in the relative expressions at the exonic and intronic levels, providing hints toward the ecological adaptation of the genus. Giant ragweed shows various levels of glyphosate resistance, with introns displaying higher expression patterns at resistant time points after the assumed herbicide treatment.

## Introduction

Plant invasions are accelerating on a global scale and cannot be fully understood without analyzing the genetic background of the source and invading populations. Biological invasions may threaten both global and local biodiversity, ecosystem functions, agriculture, and public health (Vitousek et al., 1997). Human-induced global climate change might further complicate the effects of the invasions. In Europe, as in many other regions of the world, the number of invasive plant species has increased considerably in the past 200 years due to increased trade, tourism, and disturbance (Pyšek et al., 2009). Europe suffers from invasive species in many ways, and a crude estimate of monetary impact (diminished yield and control measures) suggests that these additional costs due to invasive species exceed €12 billion annually (Kettunen et al., 2009), although this may be an underestimate (Vilá et al., 2010).

The genus *Ambrosia* L. (ragweeds) of the Asteraceae (tribe Heliantheae, subtribe Ambrosiinae) includes 40–50 species (Payne, 1964; Martin et al., 2018). Most are dioecious desert shrubs, both annual and perennial, while some species are weedy pioneers that have become established as exotics outside of their original range (Martin et al., 2018). Based on their current diversity and distribution, *Ambrosia* most likely evolved in the SW USA and adjacent Mexico, then subsequently radiated to many areas of North America (Payne, 1964). Giant ragweed (*Ambrosia trifida* L.) has raised awareness as an invasive plant in Europe — together with its relatives, common (*Ambrosia artemisiifolia* L.) and perennial ragweed (*A. psilostachya* DC.), it represents an example of aggressive invasion all over the continent. Ragweeds are among the most economically destructive weeds, as they interfere with the growth and establishment of various crops (Cowborough et al., 2003; Kong et al., 2007). *A. trifida* was primarily a weed of floodplains and ditch banks, but in the past decades it has expanded its native range in North America, causing considerable economic loss in the Corn Belt (Regnier et al., 2016). It can dominate in common croplands due to its rapid growth and large leaf area. Notably, its ability to adjust its resource utilization responses and extend its germination period has allowed it to tolerate changing environments, adding to its success as an invasive species (Abul-fatih and Bazzaz, 1979). Ragweeds also produce large amounts of pollen that causes allergenic rhinitis, presenting a burden to public health (Kazinczi et al., 2008). Currently half of all hay fever cases in North America are caused by ragweeds (Taramarcaz et al., 2005), making it one of the most powerful aeroallergens. In fact, it has been estimated that areas in Europe affected by severe ragweed allergy are likely to increase substantially by the year 2100 (Rasmussen et al., 2017). Pollen production of these plants is enhanced by special anemophily-specialized floral organs, including the pistillodium (thought to be universal within *Ambrosia*), which is a vestigial pistil in staminate flowers that forces pollen away from the corolla (Payne, 1963; Martin et al., 2009; Martin et al., 2010).

Giant ragweed is often controlled with the broad-spectrum herbicide glyphosate [*N*-(phosphonomethyl) glycine], which has become a dominant herbicide worldwide since its introduction in 1974. Although glyphosate belongs to the herbicide group with the greatest increase in resistance cases, it is still the most widely used non-selective systemic herbicide worldwide (Bracamonte et al., 2018), despite its debated environmental effects (Vandenberg et al., 2017). Recently, it was shown that glyphosate affects the gut microbiome of honeybees, inducing susceptibility to infections and death from pathogenic bacteria (Motta et al., 2018). The continuous application of glyphosate, along with reduced use of other weed management practices has also caused many weeds, including *A. trifida*, to become glyphosate-resistant (Ganie et al., 2017). Since the introduction of transgenic glyphosate-resistant (GR) crops in 1996, 19 weeds have evolved resistance to glyphosate; about half evolved in GR crops (Heap, 2018). In 2016, the area of transgenic crops reached 185.1 million ha globally, and approximately 80% were planted solely with GR crops (International Service for the Acquisition of Agri-biotech Applications [ISAAA], 2016). This means that growers continually applied glyphosate alone over these vast areas to control genetically variable and prolific weeds. The high initial efficacy of glyphosate often leads to a decline in the use of other herbicide options and less investment by industry to discover new herbicide active ingredients (Green and Owen, 2011). No matter how effective the herbicide is, weed management programs cannot rely heavily on only one tactic otherwise weeds will ultimately adapt and survive in large numbers (Green and Owen, 2011).

Our knowledge about glyphosate resistance is still limited (Duke and Powles, 2008; Sammons and Gaines, 2014), and several mechanisms have been reported in various weeds (Norsworthy et al., 2011). A specific type of resistance, called target-site resistance, may occur when single or several mutations take place in the conserved region of the EPSPS gene and/or its duplications (Yu et al., 2015; Patterson et al., 2018; Sammons et al., 2018). This gene codes the 5-enolpyruvylshikimate-3-phosphate synthase (EPSPS) enzyme that is inactivated by glyphosate, thus disrupting aromatic amino acid synthesis in plants. Such target-site resistance appears rapidly in weed populations. However, the evaluation of several glyphosate-resistant *A. trifida* accessions revealed no changes in the EPSPS gene sequence and copy number. Rather, a form of non-target-site resistance (NTSR) known as metabolic resistance has been suggested (Moretti et al., 2018; Van Horn et al., 2018). This type of metabolic resistance involves more changes than just the target site alone. Plants with NTSR rapidly metabolize or break down the herbicide before it is able to cause toxic effects (Sammons and Gaines, 2014), thus representing a major issue for the chemical control of weeds. NTSR is hard to manage because weeds have unpredictable resistance to herbicides with different chemical structures and/or target proteins (Délye, 2013). Furthermore, recent studies have demonstrated that weeds with NTSR can transmit cross-resistance to other herbicides with different modes of action, even to those not yet marketed (Petit et al., 2010). This is because NTSR is much more of a general adaptive response to herbicides (Yuan et al., 2007). According to the current view, herbicide application is a stress to the plant, which triggers response pathways in weeds irrespective of their sensitivity to the herbicide (Délye, 2013). These stress–response pathways are pre-existent in plant species, coded by multiple genes and alleles. The selection of these genes starts with the uneven application of the herbicide in the field. For example, some individuals may survive herbicide spraying because they receive less of the chemical, hence their genes are carried to future generations, providing resistance to some degree. Further NTSR evolution likely requires several generations of sexual recombination within a population until individuals accumulate enough alleles to possess a resistance level that would enable survival of the full dose of herbicide (Délye et al., 2013). Recurrent selection experiments have convincingly provided support for this theory (Neve and Powels, 2005; Busi et al., 2012). Studies of the genetic basis of NTSR are sparse, and only the key role of glutathione transferase genes have been investigated (Cummins et al., 2013). Research addressing NTSR in weeds has long been hampered by the absence of ‘omics’ resources for the vast majority of weed species. The increasing accessibility of genomics and transcriptomics supported by next-generation sequencing (NGS) technologies should rapidly enable the identification of genes governing NTSR (Délye et al., 2013). For such purposes, transcriptomes are already available for various weeds (Peng et al., 2010; Riggins et al., 2010; Zhang et al., 2012) including *A. artemisiifolia* (Taller et al., 2016b; Virág et al., 2016).

Genetic research of ragweeds has mostly been focused on *A. artemisiifolia*, with the use of microsatellite markers to determine the origins of invading populations (Genton et al., 2005a,b; Gaudeul et al., 2011). Population genetics of *A. artemisiifolia* has also been investigated (Cseh and Taller, 2008; Chun et al., 2010; Gladieux et al., 2011; Mátyás et al., 2012; Martin et al., 2016; von Boheemen et al., 2017), and genomic tools are under development for the analysis of historical specimens (Sánchez Barreiro et al., 2017) and fresh plant material (Cseh et al., 2009; Cseh, 2010; Mátyás et al., 2011; Taller et al., 2016a; Virág et al., 2016; Nagy et al., 2017). The history of common ragweed distribution was revealed based on comprehensive studies of herbarium specimens (Chauvel et al., 2006; Csontos et al., 2010; Martin et al., 2014). However, the genetics of other ragweed species in Europe have been less studied. As such, the relationships of ragweeds are poorly understood and thus a taxonomic update of Payne (1964), with newly described species and a solid phylogenetic framework of the genus is necessary. Toward this goal, herbarium specimens accumulated over the past 100 years that are readily available for sampling would be valuable material to assess ragweed diversity, and to perform evolutionary studies. Accordingly, a recent study by Martin et al. (2018) demonstrated the utility of herbarium specimens in phylogenetic analyses.

Until recently, the use of herbarium collections for obtaining molecular-level data has been difficult due to the generally poor condition of their DNA (Besnard et al., 2014). However, advances in NGS technologies and subsequent development of techniques for extracting historical DNA (Gutaker et al., 2017; Shepherd, 2017) have recently made herbarium specimens an attractive option. In fact, most NGS methods are designed for using short fragmented DNA molecules (100–400 bp) as templates, which suits herbarium specimens (Staats et al., 2013). Gutaker and Burbano (2017) demonstrated that NGS is better suited than PCR-based approaches for ancient DNA sequencing, because adapter ligation allows interrogation of the molecule ends so that longer molecules can be obtained for sequencing. As such, this approach has enabled access to genetic information on type or other important historical specimens, which are crucial for resolving taxonomic uncertainties. Moreover, NGS is both feasible and cost-effective (Bakker et al., 2015; Bakker, 2017). Staats et al. (2011) showed that it is also possible to use genome skimming to analyze DNA that is otherwise too degraded for PCR-based approaches, thus offering the possibility to include rare and extinct species from natural history collections in phylogenetic analyses. Several phylogenomic studies have explored the potential of herbarium specimens of different ages (Staats et al., 2013; Besnard et al., 2014; Aubriot et al., 2018; McManus et al., 2018; Zeng et al., 2018). For example, the entire nuclear genome of a 43-year-old *Arabidopsis thaliana* (L.) Heynh. (Brassicaceae) herbarium specimen with high and uniform sequence coverage has been reported using a reference-based assembly approach (Staats et al., 2013). NGS techniques can also be used to further acquire mitochondrial genomic data, which provides the possibility to address questions related to hybridization and introgression (e.g., Rydin et al., 2017; Vargas et al., 2017), as well as assess the phylogenetic congruence between topologies based on mitochondrial regions and plastomes (Van de Paer et al., 2018). For example, the evolutionary history of an extinct plant lineage *Hesperelaea* A. Gray, known from an 1875 collection from Guadalupe Island, has been revealed based on both the plastome (Zedane et al., 2016) and mitogenome (Van de Paer et al., 2016).

Herbarium specimens also offer a unique opportunity to study the plastid genome compartments preserved in specimens. Plastomes of angiosperms are small (typically approximately 120–190 kb in size) and have a highly conserved quadripartite structure containing two inverted repeats (IRa and IRb), which separate the large and small single-copy regions (LSC and SSC). The plastid genome includes 110 to 130 genes that participate primarily in photosynthesis, transcription, and translation (Palmer, 1985; Olmstead and Palmer, 1994; Daniell et al., 2016). Their conserved gene content, order, and organization make plastid genomes fairly well-suited for studies about gene loss, structural rearrangement, pseudogenes, or additional mutation events that might be characteristic of some lineages (Poczai and Hyvönen, 2013, 2017; Beck and Semple, 2015; Wicke and Schneeweiss, 2015). Herbarium specimens appear to yield enough reads for effective plastome assembly, similar to fresh specimens (Bakker et al., 2015). This is because plant cell walls are composed of cellulose microfibrils (Cosgrove, 1999) that provide additional protection for preservation of the genomic DNA (Bakker, 2017). The total assembly length of plastid genomes is comparable between fresh material and herbarium specimens. Those from herbarium specimens simply need slightly more editing and “scaffolding” because on average they yield shorter contigs (Bakker et al., 2015; Bakker, 2017; Twyford and Ness, 2017).

In the current study, we sought to develop further resources to facilitate genomic research of ragweeds. By using a herbarium specimen, we aimed to demonstrate the utility of such collections for weed genomics. We present the complete chloroplast genome sequence of giant ragweed (*A. trifida*) using high-throughput sequencing, and report the assembly, annotation, gene expression, and unique structure characterization of its plastome. Comparison of the gene order and inverted repeat (IR) length across Asteraceae is also presented, as well as transcriptional profiling against glyphosate resistance.

## Materials and Methods

### DNA Isolation and Plastome Sequencing

Giant ragweed leaves (0.5 g) were obtained from a herbarium specimen of the Finnish Museum of Natural History (H), University of Helsinki, Finland (H1645542; Mansfield, OH, United States, 1886). Leaf samples were rinsed with deionized water and 70% ethanol, and total genomic DNA was isolated using the NucleoSpin Plant II kit (Macherey-Nagel, Düren, Germany). All work was carried out in a dedicated laboratory with UV sterilized equipment; blank samples were processed during DNA extraction. DNA concentration was measured with a Qubit fluorometer (Invitrogen) and verified on an 0.8% agarose gel. A paired-end genomic library was constructed using the Nextera DNA library preparation kit (Illumina, San Diego, CA, United States). Fragment analysis was conducted with an Agilent Technologies 2100 Bioanalyzer using a DNA 1000 chip. Sequencing was performed on an Illumina MiSeq platform from both ends with 150 bp read length.

### Genome Assembly and Annotation

To obtain high-quality clean data, raw reads were first filtered by removing low-quality reads with a sliding window quality cutoff of Q20 using Trimmomatic (Bolger et al., 2014). Plastid reads were obtained by reference mapping using *A. artemisiifolia* L. (MG019037; NC035875) plastid genome (Amiryousefi et al., 2017; Nagy et al., 2017) with Geneious R10 (Kearse et al., 2012). Clean reads were used for *de novo* assembly, which was performed with three different algorithms of the programs Velvet v1.2.10 (Zerbino and Birney, 2008), Geneious assembler, and NOVOPlasty (Dierckxsens et al., 2017). General genome structure and ambiguous nucleotide positions were evaluated by an additional assessment. Sequence inconsistencies were checked by mapping reads to the plastome as described in Wysocki et al. (2015). The final *de novo* assembly was used to map the reads and calculate the mean coverage of the plastid genome using Geneious R10. Sanger-based gap closure and IR junction verification was performed following Poczai and Hyvönen (2017). Annotation was performed using a two-step procedure. First, we transferred all annotations in Geneious from the reference *A. artemisiifolia* to the *A. trifida* genome using a similarity threshold of 80%. The genome sequence was also annotated using GeSeq (Tillich et al., 2017), since this program performed better than other algorithms in a comparative study (Amiryousefi et al., 2018b). In a second step, we inspected, compared, and curated all annotations manually. This included extracting all coding regions per genome, confirming start/stop codons and features for each gene, and aligning extracted regions across all plastid genomes to confirm approximate gene lengths based on their conserved gene order. Final curated annotations were transferred to the complete plastid genome sequence of *A. trifida*, which was deposited in GenBank (NC036810). The resulting genome map was drawn with OGDraw v.1.2 (Lohse et al., 2007).

### Comparative Plastome Analysis of Sequenced Asteraceae With *Ambrosia trifida*

To highlight the significance of the sequenced *A. trifida* plastome compared to the previously available plastomes of Asteraceae, we first investigated the repeat proportion of the genome using MISA (Thiel et al., 2003; Beier et al., 2017) and REPUTER (Kurtz et al., 2001) to identify the stretches of the perfect, compound, and long-forward repeats. MISA was used to analyze the perfect microsatellites [often abbreviated as simple sequence repeats (SSRs)] with a defined length of *n* = 10 in mononucleotide repeats, *n* = 6 in dinucleotide repeats, and *n* = 3 in tri-, tetra-, penta-, and hexa-nucleotide repeats. For the compound repeats, two defined SSRs should be interrupted by 100 bp. For comparative analysis, a repeat profile was mined across the 10 species to observe any divergence in the occurrence of the repeat motifs. For the identification of the forward repeats, REPUTER was used with a defined length of 30 bp and a hamming distance of 3. For the identification of microstructural events, pairwise alignments of the Asteraceae plastomes with *A. trifida* were performed using LASTZ (Harris, 2007) and MuMMER version 3.1 (Kurtz et al., 2004). The show-snps feature was used to evaluate the identification of plastomic variations. Once microstructural events were identified, they were further plotted using Circos (Krzywinski et al., 2009) and ggbio (Yin et al., 2012).

### Comparative Phylogenomics

We based our sampling on the results of Panéro and Funk (2002), updated in Panéro et al. (2014), considering the availability of currently deposited plastid genomes in the Organelle Genome Resources of NCBI (Wolfsberg et al., 2001) accessed on 12.12.2017. Of the currently accepted 13 subfamilies of Asteraceae, only three (Carduoideae [8], Cichorioideae [8], and Asteroideae [128]) had complete plastid genome sequences deposited with a prominent bias in the genomes of Asteroideae. Genomes within subfamilies were chosen to represent each (super)tribe from the available sequences. For Carduoideae, we included one species from all accessible genera that were sequenced and available at the time we conducted the phylogenetic analysis. In Cichorioideae, only two — *Lactuca* L. and *Taraxacum* F. H. Wigg — were available. From Asteroideae, we aimed to include at least one species from each of the 21 tribes. We also included genome sequences of *Carum carvi* L. and *Foeniculum vulgare* Mill. of Apiaceae from the campanulid clade (Angiosperm Phylogeny Group, 2016) as outgroups. Accession numbers are provided in Supplementary Table S1. For phylogenetic analysis, we used a matrix of 50 protein-coding genes representing 43 species, with a total concatenated matrix alignment of 31,356 bp. We estimated congruence between different sources of information by comparing the whole genome alignment matrix, coding region matrix, and non-coding region matrix (intergenic regions). We observed ambiguity in the repeat expansion, which might affect the gene composition. Since large microstructural variations such as single nucleotide polymorphism (SNPs), and insertions/deletions (INDELs) were observed, phylogenetic analyses were limited to the coding regions (Curci et al., 2015).

From all the sequenced Asteraceae and *A. trifida*, coding alignments were constructed for the following fifty protein-coding genes: *cemA, infA, matK, ndhC, ndhD, ndhE, ndhF, ndhG, ndhH, ndhI, ndhJ, ndhK, petA, petG, petL, psaA, psaB, psaC, psaI, psaJ, psbA, psbB, psbC, psbD, psbF, psbH, psbI, psbJ, psbK, psbL, psbM, psbN, psbT, rbcL, rpl14, rpl20, rpl22, rpl32, rpl33, rpl36, rpoA, rpoB, rps2, rps3, rps4, rps8, rps11, rps14, rps18*, and *ycf4*, using MACSE (Ranwez et al., 2011), which uses a frameshift alignment algorithm for aligning coding sequences. Following the MACSE alignments, each frameshift was masked and the subsequent alignment was trimmed using trimAL (Capella-Gutiérrez et al., 2009). Finally, prior to the construction of the super-matrix, terminal stop codons were identified and subsequently removed from the trimmed alignments. Concatenation of the phylogenetic matrix was performed using SequenceMatrix version 1.8 (Vaidya et al., 2011). The concatenated sequence matrix was analyzed by maximum likelihood (ML) and parsimony methods. ML analyses were performed with IQ-TREE (Nguyen et al., 2015). The best fitting model (GY+F+I+G4) was determined by ModelFinder (Kalyaanamoorthy et al., 2017) as implemented in IQ-TREE according to the Akaike information criterion (AIC), and Bayesian information criterion (BIC). To assess branch support, all IQ-TREE analyses used the ultrafast bootstrap approximation (UFBoot; Hoang et al., 2018) with 1,000 replicates and the SH-like approximate likelihood ratio test (SH-aLRT) also with 1,000 bootstrap replicates.

Parsimony analyses were performed using nona (Goloboff, 1994) within winclada (Nixon, 2002) shell. Prior to analyses, the command “mop uninformative characters” was used to exclude parsimony uninformative characters. This resulted in a matrix of 3,769 characters. Two separate searches were performed (using processor time as a seed to randomize the order of the terminals) with the following settings: hold 30,000 (holding defined maximum number of trees), 100 replications (search performed with multiple tree-bisection-reconnection algorithm mult^∗^max^∗^), hold/3 (keeping three starting trees for each replication). In addition, we also performed larger analyses by keeping 20 starting trees for each replication (hold/20) with 1,000 replications. To assess whether the longer genes have any compositional heterogeneity, we created a partition-specific matrix of the long genes *ndh*D, *ndh*H, *psa*A, *psa*B, *psb*A, *psb*B, *psb*C, and *rbc*L and evaluated the skewness and the compositional heterogeneity across the combined and partition-specific variations.

To reveal the placement of the genes near the inverted repeat junction sites of *A. trifida*, an IR plot of this species and nine other Asteraceae were obtained with IRscope (Amiryousefi et al., 2018a).

### Transcriptome Profiling

Despite their small size, plastids represent a classical example of miniature genomes containing mono- and polycistronic transcripts. With the advent of plastome sequencing, there has been considerable interest in understanding the transcriptional activity of plastid genes, as well as other events related to RNA editing and post-transcriptional splicing. To understand the transcriptional divergence associated with glyphosate resistance, which is defined as the amount of the transcriptional plasticity between the sensitive and resistant treatment in the giant ragweed plastome, we mapped the RNA-seq reads previously deposited to the currently sequenced *A. trifida* plastid genome using ChloroSeq (Castandet et al., 2016), with the defined exonic and intronic localization in the annotated GFF3 (NCBI SRA: PRJNA267208; Supplementary Table S2). For mapping the RNA-seq reads, tophat2 (Kim et al., 2013) was used with -g 2 and -no-novel-junctions functions to minimize the identification of the novel splice sites. IR repeat diversity, mapping read estimation, and genome coverage was subsequently calculated using SAMtools version 1.15 (Li et al., 2009) and bedtools version 2.25 (Quinlan and Hall, 2010). Expression values in terms of the RPKM values were mapped to the genome features and were visualized using Circos (Krzywinski et al., 2009) and ggbio (Yin et al., 2012).

## Results and Discussion

### Genome Assembly and Plastome Features

**FIGURE 1:**
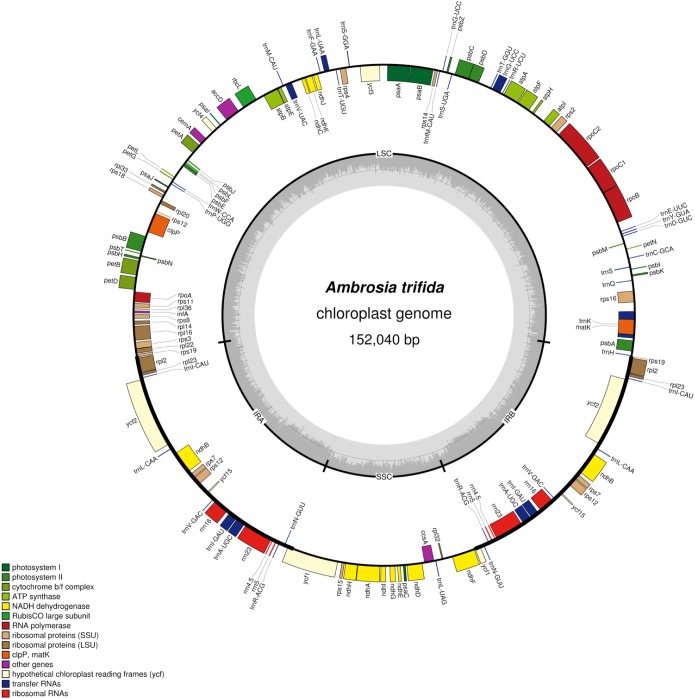
Plastome map of *Ambrosia trifida*. Gene features and distribution of the direction of genes with respect to their localization. Genes on the inner side of the outer circle are transcribed counterclockwise while those on the outer side are transcribed clockwise. Genes belonging to different functional groups are color-coded; the GC and AT content of the genome are plotted on the inner circle as dark and light gray, respectively. Inverted repeats (IR); large single copy (LSC) regions; small single copy (SSC) regions.

Plastid DNA sequencing generated 145,207 paired-end reads, with an average fragment length of 267 bp. *De novo* assemblies of reads resulted a total of 38 contigs with an N50 of 13,231 bp. Ten contigs were alignable and covered 100% of the *A. artemisiifolia* reference genome. Mapping of the reads to the *de novo* assembled plastid genome resulted in a 180× mean coverage. The chloroplast genome of *A. trifida* was 152,040 bp and showed a quadripartite structure of long (83,966 bp) and small (17,894 bp) single-copy regions, separated by two inverted repeat regions of 25,090 bp (Figure 1). As in other species of Asteraceae, the *A. trifida* chloroplast genome contains 80 protein-coding, 28 tRNA, and four rRNA genes comprising a total of 112 unique genes (Supplementary Tables S3, S4). The distribution of the genes also exhibited similarity with other Asteraceae and angiosperms, with 13 genes found in the SSC, 19 genes in the IR, and 80 genes in the LSC. The overall GC content of the chloroplast genome was 37.2%. Only 21% of the whole genome is non-coding. There were 18 intron-containing genes in the giant ragweed plastome (Supplementary Table S4). From these, 16 (10 protein-coding and six tRNA) genes had a single intron, and two (*ycf*3, *clp*P) had two introns. 12 (eight protein-coding and four tRNA) genes are located in the LSC, one (protein-coding) gene in the SSC, and five (three protein-coding and two tRNA) genes in the IR region. The largest intron (2,565 bp) was located in the *trn*K-UUU gene, including the highly diverse *mat*K gene. The *trn*K intron is of interest because it represents an unusual form of a group II intron derived from a mobile group of mitochondrial-like intron ORFs (Hausner et al., 2006). The *rps*12 gene was trans-spliced with the 5′ end exon located in the LSC region and the two remaining exons found in the IR regions. We also observed three cases of overlapping genes, namely *psb*D/*psb*C, *atp*E/*atp*B, and *rps*3/*rpl*22. In the *ndh*D and *psb*L genes, we observed that ACG is used as an alternative start codon instead of the common AUG in *A. trifida* and in most species of Asteraceae. It has been shown that this exceptional ACG start codon is RNA edited in all Solanaceae (Amiryousefi et al., 2018b) except *Datura stramonium* L., while the start codon of *psb*L is unedited. In Asteraceae, the canonical AUG form is found for both *psb*L and *ndh*D in *Ageratina adenophora* (Spreng.) King & H. Rob., *Pericallis hybrida* (Willd.) R. Nordenstam, *Silybum marianum* (L.) Gaertn. However, only *ndh*D possesses the AUG start codon in *Centaurea diffusa* Lam., *Chrysanthemum indicum* L., *Jacobaea vulgaris* Gaertn., *Mikania micrantha* Kunth and *Saussurea involucrata* Matsum. & Koidz.

### Genomic Repeats and Rearrangements

Microsatellites (or SSRs) are valuable molecular markers of high-degree variations within the same species. These markers have been previously used in population genetics and polymorphism investigation of ragweeds (Genton et al., 2005a,b; Gaudeul et al., 2011). We analyzed the distribution of SSRs according to the defined length criteria in the giant ragweed plastome. A total of 100 SSRs were observed, with eight present in compound form. To understand whether their distribution varies among Asteraceae species, we further compared 10 previously sequenced plastomes (Table 1). The most abundant motifs of the SSRs were poly-A/T stretches characteristic of angiosperm plastid genomes. These results are consistent with previous findings that the SRs are generally composed of short poly-A or poly-T repeats and rarely contain tandem G or C repeats h. In addition to mononucleotide stretches, we observed tetra- (AGAT/ATCT) and hexa- (AAGGAT/ATCCTT) nucleotide repeats, which could be of specific interest for future cpSSR marker development in cross-species amplifications or population genetic studies of ragweeds. We further identified 25 larger repeats (>30 bp) using the defined parameters in REPUTER (Kurtz et al., 2001). Most of these long repeats were present in the intergenic spacers (Table 2). Among these repeats, a large portion were found within *ycf* genes, which have been shown to have high divergence rates in most embryophyte lineages and have undergone pseudogenization (Michelangeli et al., 2003). The nucleotide sequence similarity among embryophyte *ycf*2 is extraordinarily low compared to other plastid-encoded genes: it is less than 50% across bryophytes, ferns, and seed plants (Wicke et al., 2011). This divergence is not surprising since *ycf* genes have experienced many insertions/deletions. For example, these deletions account for the reduction in the chloroplast genome size among members of the Graminid clade (Poczai and Hyvönen, 2017).

**Table 1:** Distribution and summary of the shared SSRs across the Asteraceae plastomes.

Repeats	*Ambrosia trifida*	*Helianthus annuus*	*Jacobaea vulgaris*	*Taraxacum officinale*	*Echinacea purpurea*	*Artemisia annua*	*Soliva sessilis*	*Westoniella kohkemperi*	*Carthamus tinctorius*	*Ambrosia artemisiifolia*
A/T	30	39	34	19	38	39	16	49	19	36
AAC/GTT	4	4	4	4	4	4	4	4	3	4
AAG/CTT	28	24	24	20	25	21	23	22	20	28
AAT/ATT	16	18	26	20	19	26	34	40	24	19
ACC/GGT	2	3	NIL	2	3	3	3	3	3	2
ACG/CGT	1	1	1	1	1	NIL	NIL	1	1	1
ACT/AGT	1	1	1	1	1	NIL	2	NIL	NIL	1
AGC/CTG	7	7	6	7	7	7	7	7	6	7
AGG/CCT	3	3	4	2	2	4	3	3	3	3
ATC/ATG	3	3	3	3	3	5	4	3	3	3
AAAG/CTTT	1	1	1	3	1	1	1	NIL	1	1
AAAT/ATTT	2	2	5	1	2	7	3	9	3	4
AGAT/ATCT	1	1	NIL	NIL	1	NIL	NIL	2	NIL	1
AAGGAT/ATCCTT	1	NIL	NIL	NIL	1	NIL	NIL	NIL	NIL	1


**Table 2:** Summary of the identified forward repeat stretched across the *Ambrosia trifida* plastome.

No	Type	Location		Region	Repeat unit	Period size (bp)	Copy nr.
1	P	*ycf*3/*ndh*A	Intron	LSC/SSC	CAGAACCGTACATGAGATTTTCATCTCATACGGCTCCTC	41	2
2	P	*rps*12-*ycf*16	IGS	IR	CAGAACCGTACATGAGATTTTCA[CT]CTCATACGGCTCCTC	39	2
3	P	*rps*12-*ycf*15	IGS	IR	TATTAGATTAGTCTATTAATTCATATTAGATTAGTCT	37	2
4	F	*pdb*E – *pet*L	IGS	LSC	ATTCATGAATTGATTAGAATATTGCCGCAATTG	34	2
5	F	*ycf*2	Gene	IR	TGACGATATTGATGCTAGTGACGATAT	27	2
6	P	*psb*T-*psb*N	IGS	LSC	AATTGAAGTAATGAGCCTCCCAAT	24	2
7	F	*rps*12-*ycf*15	IGS	IR	CTATTAGATTAGTCTATTAATTCA	23	2
8	F	*rpl*32-*ndh*F	IGS	SSC	ATAAAAATATTCAATAAGTATAA	23	2
9	F	*trn*D – *trn*Y	IGS	LSC	TTCTCTCGTATCAGGTAT	18	3
10	F	*ycf*1	Gene	SSC	AATGGAAATAGAAGAAG	18	3
11	F	*psb*K -*psb*I	IGS	LSC	ATACCTTATTAGC	13	4


*IGS, intergenic spacer; IR, inverted repeat; LSC, large single copy region; SSC, small single copy region; F, forward repeat; P, palindromic repeat.*

**FIGURE 2:**
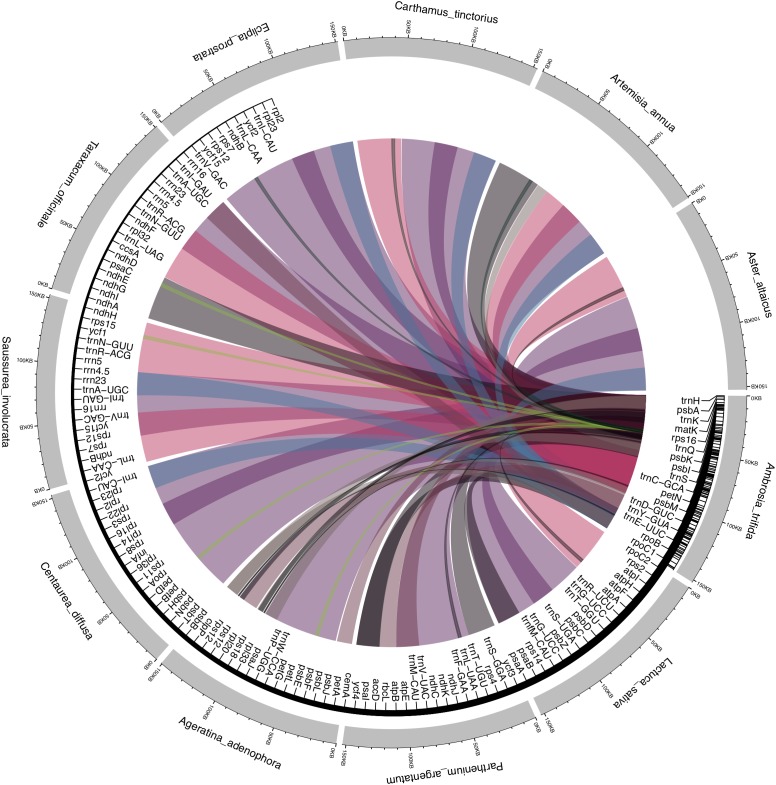
Segmental synteny and conservation of *Ambrosia trifida* across Asteraceae plastomes. LASTz alignments were filtered to keep only colored tracks showing > 70% alignment coverage, with the colored ribbons are species-specific. The inner tract displays the corresponding gene annotations of *A. trifida*, with the corresponding ribbons showing the inversions and segmental rearrangement across the gene regions.

Asteraceae plastid genomes contain two inversions of 22.8 and 3.3 kb as compared with the outgroup terminals *Carum carvi* and *Foeniculum vulgare* of Apiaceae. The larger inversion is located between the *trn*S-GCU and *trn*G-UCC genes, and the smaller between the *trn*E-UUC and *trn*T-GGU genes. In addition to these large inversions, another smaller inversion of 3.3 kb is located within the larger inversion, between the *trn*C-GCA and *rpo*B genes. These rearrangements are assumed to have originated in the late Eocene (36–42 My BP) and are commonly reported for Asteraceae (Jansen and Palmer, 1987; Kim et al., 2005). They are absent in the species of the basal subfamily Barnadesioideae as assessed by restriction endonuclease digestions (Jansen and Palmer, 1987). This interesting finding should be confirmed by future research, since complete plastid genome data is currently missing for this group. To further understand the level of the microstructural events, we performed a pairwise comparison of the Asteraceae plastomes with the sequenced *A. trifida*, revealing a high number of substitutions compared to insertions and deletions. Interestingly, we observed a large number of inversions compared with *Parthenium argentatum* A. Gray and *Ageratina adenophora* (Spreng.) King & H. RobAs in other angiosperms, the coding regions of Asteraceae are more conservative than the non-coding regions; *rpo*C1 is the most divergent of all the genes. The invasive weed *Ageratina adenophora rpo*C1 contains two introns, while only one intron is found in the plastid genomes of other Asteraceae (Nie et al., 2012). Our LASTZ dot-plot comparison with the complete plastome of *Saussurea involucrata* Matsum. & Koidz. showed rearrangement patterns (Figure 2 and Supplementary Figure S1) caused by a large shift (approximately 93 kb) in residue numbering that caused problems during de-circularization of the genome. The published genome of this species (Xie et al., 2015) also contains numerous annotation errors with several key genes unannotated (for example, *rpl*16 exon 2, *rps*12, and truncated *rps*19). We found that SSC regions showed signatures of inversions (Figure 2), which is consistent with previous reports (Liu et al., 2013; Walker et al., 2014; Gruenstaeudl et al., 2017). These major spots of inversions have also been reported previously in the plastomes of several plant lineages (Schwarz et al., 2015; Hsu et al., 2016; Graham et al., 2017; Sinn et al., 2018), It is important to note that the SSC region occurs in two inversion isomers that exist in equimolar proportions in the same individual, which are identical in sequence but different in orientation (Palmer, 1983). Presently, whole plastid genome sequences are uploaded in GenBank without preference for the orientation of the SSC region, which appears as a genome structural inversion but is truly just chloroplast heteroplasmy (Walker et al., 2015). Many plastid genome studies use reference-guided mapping, where the orientation of the SSC is copied along novel genomes. For example, SSC regions occur in inverted orientations in Solanaceae compared to Asteraceae (Salih et al., 2017). Another good example in Asteraceae is the plastid genome of *Lactuca sativa* L., which has been published twice independently (NC007578 and DQ383816). Besides minor polymorphisms among these genomes, the major difference is the orientation of the SSC, which appears to be inverted. Due to the rapid increase in amount of *de novo* assembled plastid genomes that are often deposited independently and parallel to each other, this phenomenon should not be overlooked, and SSC orientations should not be regarded as diversity hot-spots.

### Inverted Repeat (IR) Diversity

**FIGURE 3:**
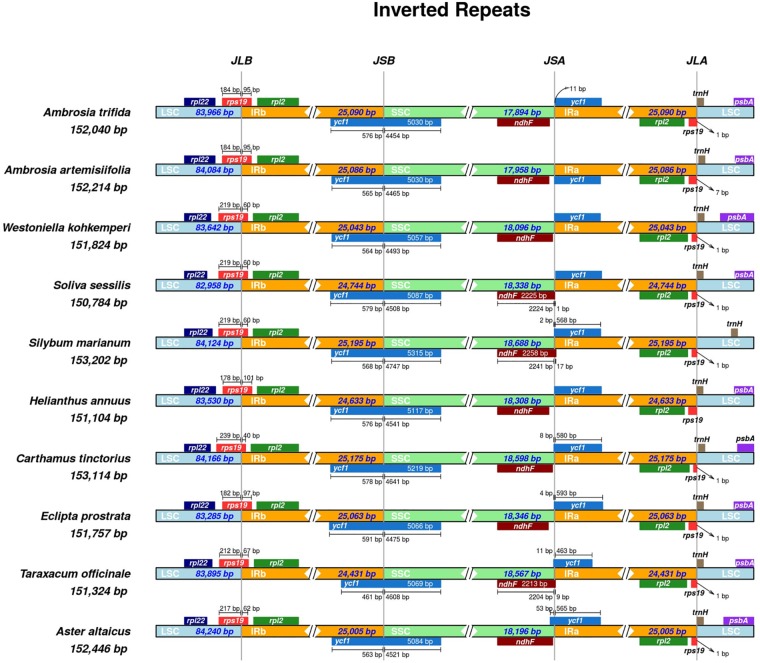
Inverted repeat plot of *Ambrosia trifida*. *rps*19 gene on the LSC/IRb regions with corresponding 184/95 bp. The positive strand synteny of the *rpl*2, *rps*19, and *rpl*22 on the IRb, JLB, and LSC, respectively, is also confirmed. In accordance with the other selected nine Asteraceae, the JSB of *A. trifida* is hosting the extended functional version of the *ycf*1 gene, while the pseudo version of this gene beside *ndh*F is placed near the JSA site. Another copy of the *rps*19 and *trn*H, along *rpl*2 are gathered near the JLA.

Most of the length variation in angiosperms is attributed to expansion or contraction of the IR regions. It is rare that this variation is due to gene losses, which could vary within a single genus (Sloan et al., 2014). To obtain comprehensive insight into the IR regions of the Asteraceae, a survey of over 40 species was performed using a recently published tool IRscope (Amiryousefi et al., 2018a). This revealed the placement of *rps*19 in the JLB (LSC/IRb) in almost all cases (Figure 3 and Supplementary Figure S2). The inversion of the SSC region, and hence its reversed gene annotation, is a main distinguishing factor in the visual representation of the species. The fixation of the extended *ycf*1 in the JSB (IRb/SSC) was confirmed for the majority of the species (approximately 80%). For the same set of species, we observed a pseudo *ycf*1 gene often tangental to the JSA (SSC/IRa) on the IRa side and the *ndh*F gene near the JSA on the SSC side. In some species, the SSC occurred in the reverse orientation and with *ycf*1 extending on the JSA site and a pseudo *ycf*1 and *ndh*F plotted on the JSB. The JLA has the *rps*19 and *trn*H genes in its vicinity, with *psb*A further in the LSC region in almost all cases. The pseudo *rps19* gene was unannotated in the original files of the first four species in Figure 3. Hence, we obtained the corresponding annotations using GeSeq and inverted the SSC region with IRscope for the first five species to improve visual comparison. Unlike the others, the new annotation of *A. artemisiifolia* confirmed the existence of the *rps19* and its corresponding pseudo fragment on the LSC adjacent to IRb and IRa, respectively (Figure 3). Non-identical IR features were observed for *A. artemisiifolia* (NC035875), where the IRb contains a single C base insertion near *rpl*2 (84,242 bp). The *trn*H gene was also assigned to the opposite DNA strand, which we have manually corrected for Figure 3. After these adjustments, we obtained a corrected size of 25,086 bp for the IRs (cf. Nagy et al., 2017).

The conservation of the length of the IRs, SSC, and LSC is another interesting point. These regions were between 24 to 26, 18 to 19, and 82 to 84 kb, respectively. The maximum length of plastid genomes is 153,014 bp for *Conyza bonariensis* (L.) Cronquist, while the shortest length of 149, 510 bp was found in *Aster spathulifolius* Maxim. As in other Asteraceae, the inverted repeat plot of the *A. trifida* showed the *rps*19 in the LSC/IRb regions with a corresponding 184/95 bp. The synteny of the *rpl*2, *rps*19, and *rpl*22 (for IRb, JLB, and LSC, respectively) of the positive strand was also confirmed. As in the majority of other angiosperms, the JSB of *A. trifida* has the extended functional version of the *ycf*1 gene while the pseudo version of this gene beside *ndh*F is placed near the JSA site. Another copy of the genes *rps*19, *trn*H and *rpl*2 can be found near the JLA.

### Comparative Phylogenomics

**FIGURE 4:**
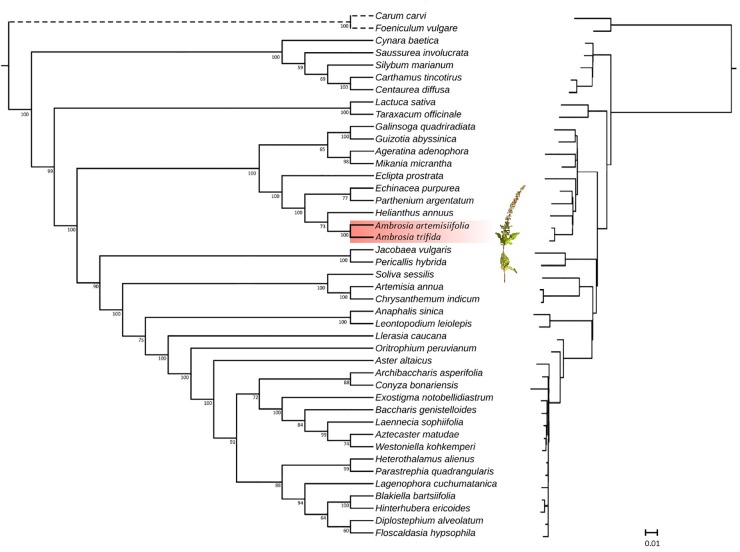
Phylogenetic tree (left in cladogram style, right with branch lengths) inferred from plastid genome data. Maximum likelihood phylogeny illustrating the phylogenetic relationships of Asteraceae based on fifty protein-coding genes. Species of the genus *Amrbosia* are highlighted in red. Branch lengths are proportional to the number of substitutions, while numbers represent bootstrap support values for each node. The given scale represents substitutions per site.

Asteraceae is one of the largest families of flowering plants, with approximately 1,500 genera and 23,000 species (Kumar et al., 2009). While several studies have been conducted to resolve the phylogeny (Denda et al., 1999; Panéro and Funk, 2002, 2008; Panéro et al., 2014), many questions remain open. Plastome sequences can now be easily acquired for phylogenomic analyses at relatively low costs, thus providing rich sources of phylogenetic information. To assess the phylogenetic position of *A. trifida* relative to the previously sequenced Asteraceae species, we performed maximum likelihood (Figure 4) and parsimony analysis. The latter resulted in six equally parsimonious trees with length of 7,297 steps, with consistency index (Kluge and Farris, 1969) CI 0.65 and retention index (Farris, 1989) RI 0.83 (Supplementary Figure S3). The tree is congruent with that obtained by using maximum likelihood as an optimality criterion, and no bias in compositional heterogeneity was detected in the sequences (Supplementary Figure S4).

Historically, Asteraceae were divided into two large subfamilies (Asteroideae and Cichorioideae) and 13 tribes (Bentham, 1873). Major changes in classification have been made during the past decade, resulting in a better phylogenetic framework. Based on the analyses of chloroplast DNA markers (Panéro and Funk, 2002, 2008; Panéro et al., 2014), 13 major clades (subfamilies) were identified in Asteraceae. From these subfamilies, our study included representatives of Carduoideae, Cichorioideae and Asteroideae with complete plastid genome sequences available from public databases. The Asteroideae subfamily was sister to Cichorioideae, including *Lactuca sativa* L. and *Taraxacum officinale* (L.) Weber ex F. H. Wigg of the tribe Cichorieae. Taxa within the subfamily Carduoideae were represented by members of the tribe Cynareae, and formed two clades. The first was composed of *Carthamus tinctorius* L. and *Centaurea diffusa* Lam. The second included *Cynara baetica* (Spreng.) Pau and *Silybum marianum* (L.) Gaertn. as sisters, while *Saussurea involucrata* Matsum. & Koidz. were resolved in a basal position. This phylogeny obtained for the Cynareae is consistent with the results of a more detailed molecular analysis (Barres et al., 2013) including larger number of terminals. As expected, our tree resolved three supertribes Asteroideae, Helianthidae, and Senecionodae within the subfamily Asteroideae. *Jacobea vulgaris* Gaertn., together with *Pericallis hybrida* (Willd.) R. Nordenstam, grouped in the supertribe Senecionodae. In the Asterodae, all three sampled tribes of Anthemidae, Gnaphalieae, and Astereae were distinct with high support values compared to previous plastid studies where tribes of Asteroideae were not resolved (Curci et al., 2015). *Helianthus annuus* L, *A. artemisiifolia,* and *A. trifida* grouped in the Heliantheae alliance indicating a group with high support values within the supertribe Helianthodae, and separate of Millerideae (*Galinsoga quadriradiata* Ruiz & Pav., *Guizotia abyssinica*) and Eupatorieae (*Ageratina adenophora* [Spreng.] King & H. Rob.; *Mikania micrantha* Kunth). Deeper level relationships within the alliance Heliantheae were unresolved. Whole chloroplast genome sequences were unavailable for the cockleburs (*Xanthium* L.) thus we were unable to provide support for the previously reported sister genus relationship to *Ambrosia* (Miao et al., 1995). Both genera are monoecious with pistilate florets surrounded by woody involucres, a morphological trait that has been assumed to indicate their close relationship (Peterson and Payne, 1973).

### Comparative Transcriptional Profiles Against Glyphosate Resistance

As a first contribution toward deciphering the complete genetic information of giant ragweed, we determined the complete sequence of its plastome. Since assembling plastid genomes from herbarium specimens is possible, the complete sequences can be used in further applications. The analysis presented here is an example of the use of herbarium genomics in other fields like weed research where, besides the biology of ragweeds, their control with herbicides is a major area of focus for research. Herbicides have a pivotal role in controlling weeds and sustaining food security by restricting weeds while being as harmless as possible for crops (Duhoux et al., 2017). Reaching this specific goal with various mechanisms of action, herbicides induce changes in gene expression to alter or terminate plant physiological pathways. Knowledge about the regulation of plastid gene expression in response to herbicide treatments is central to our understanding of photosynthesis and other plastid-localized metabolic pathways associated with herbicide resistance. To date, the molecular basis of herbicide resistance has been largely investigated by single-gene sequencing to identify single-point mutations in the target site of the herbicide. More recently, second-generation sequencing technologies have also enabled transcriptomic approaches (e.g., RNA-seq) to identify candidate genes underlying more complex NTSR mechanisms, such as herbicide metabolism and translocation (Ravet et al., 2018). Weed genomics offers the promise to go beyond transcriptomics and provide further novel insights into the biological processes such as NTSR.

**FIGURE 5:**
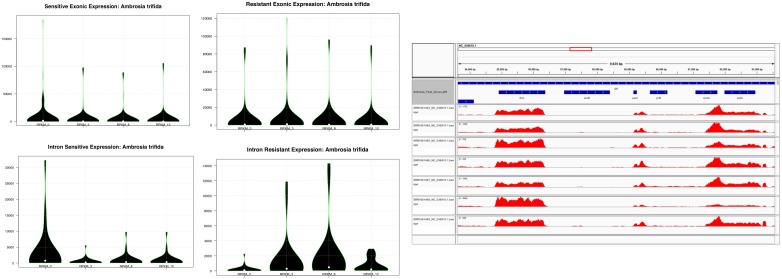
Violin plots showing the exonic and intronic RPKM distribution across the sensitive and resistant glyphosate ragweeds. Time points indicate sample collection: 0, 3, 8, and 12 h after herbicide application.

**FIGURE 6:**
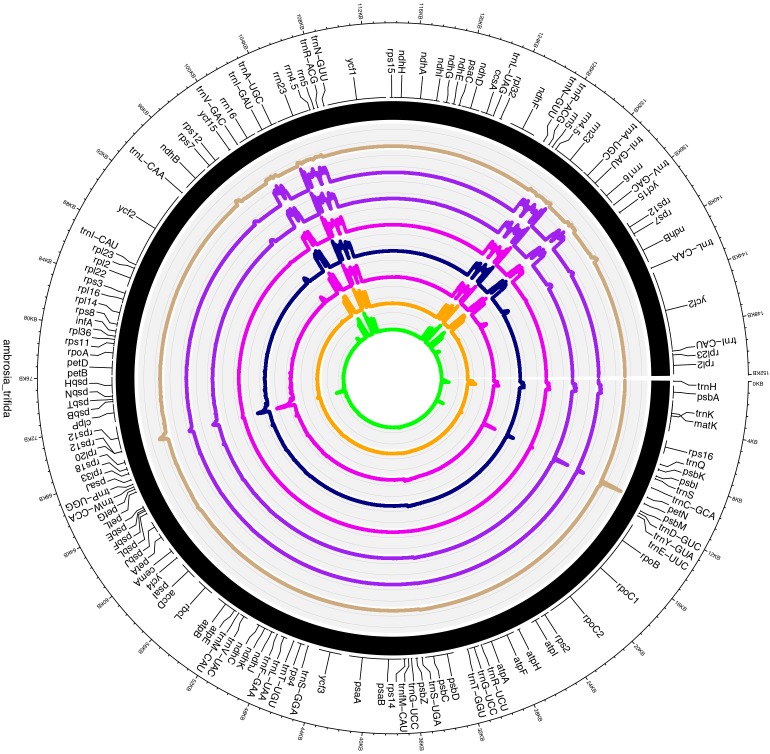
Circos plot visualization of the transcriptional mapping of *Ambrosia trifida*. Single-base resolution of the mapped RNA-seq reads across the *A. trifida* plastome with represented gene annotations. Track ribbons order according to the following SRA accession number: SRR1661420, SRR1661463, SRR1661464, SRR1661465, SRR1661466, SRR1661467, SRR1661468 and SRR1661469.

Despite its small size, the transcriptional apparatus of plastids is relatively divergent at the expression level compared to the nuclear genome, which is the first line in transcriptional-mediated responses pertaining to abiotic and biotic stress including herbicide resistance. These transcriptional events, as defined by the variations observed in the transcriptional expression levels, play an important role in understanding plastid metabolism at the cellular and energetic levels by altering the transport of solutes across the membrane, or by regulating their sequestration through membrane trafficking (Sammons and Gaines, 2014). One such example is glyphosate, which is sequestered by a transport mechanism and inhibits the shikimate pathway in the chloroplast (Sammons and Gaines, 2014). Plastid gene expression and its regulation has been intensely studied in chloroplasts (see the review of Leister et al., 2017). By contrast, knowledge of gene expression under herbicide treatment in plastids is still very limited. Since a large fraction of plastid protein-coding genes is involved in photosynthesis, it is generally believed that plastid gene expression is affected in plant tissues treated with herbicides. Until now, limited knowledge has been accumulated to understand the transcriptional events related to glyphosate resistance, although it has been used globally to control noxious weeds such as *A. trifida* (Sammons and Gaines, 2014). As previously mentioned, since glyphosate sequestration and harvesting through the chloroplast mediated shikimate pathway play an important role in herbicide resistance, we compared the transcriptional events in the plastid genome of *A. trifida.* For this, we used RNA-seq reads previously deposited in the Sequence Read Archive (Supplementary Table S2) gathered from total plant cell transcriptomes, which capture both primary and processed mRNA sequences of the plastome. Therefore, we first isolated the plastid genome data from the total transcriptomes of glyphosate resistant and sensitive *A. trifida* plants sequenced across four different time points – 0, 3, 8, and 12 h after spraying with the herbicide. In particular, we observed that exonic divergence (change in the patterns of the expression levels of exons across the sensitive and the resistant time points) to be more pronounced compared to the intronic divergence (change in the patterns of the expression levels of introns across the sensitive and the resistant time points; Figure 5 and Supplementary Table S5). Considering the divergence of the glyphosate uptake, a higher expression of plastid genes was found in resistant as compared to the sensitive conditions. As a first line of defense against any abiotic stress, the primary focus of the gene expression alternation at the nuclear level is mainly pertaining to the genes involved in photosynthetic responses, heat shock or those involved in the membrane transport. We observed a higher expression of the tRNA genes across all of the time points, which is conserved across all of the embryophytes and suggests that the higher expression of the relative tRNAs is required for rapid transcription activity, which might be required to circumvent the herbicide-mediated changes in the membrane transport of the generated ATPs across the NADPH complex. Additionally, it might indicate that the expression of the SIG2, responsible for the transcription of tRNA genes, is pre-dominant as compared to the SIG6, which is mostly associated with the photosynthetic genes (Figure 6).

Furthermore, *A. trifida* had relatively low expression levels, which might hint toward the uptake of the glyphosate and the rate of disruption of the photosynthetic apparatus and the metabolic pathways associated with plastid genes. It has been widely demonstrated that glyphosate also affects growth by contaminating the environment and accumulating in plant organs, and can be a major limiting factor for agricultural productivity (Fartyal et al., 2018). According to our observations, plastid intronic expression is higher in glyphosate-resistant giant ragweed at all sequenced time points compared to the sensitive time points after the herbicide treatment (Supplementary Table S5). The expression of introns associated with *ycf3* increased during the 8 and 12 h time points in sensitive *A. trifida*, which might present a defense mechanism to combat glyphosate (Figure 6). Relative expression divergence among the sensitive and resistant time points for the photosystem I (PSI) assembly protein *ycf3* revealed an up-regulation in the resistant phenotype compared to the sensitive phenotype. This clearly reflects the role of glyphosate as an interfering herbicide in plant growth through the inhibition of the photosynthetic complex (Fartyal et al., 2018). Higher expression of ribosomal-associated genes has also been observed in sensitive time points compared to the glyphosate resistant time points, which might reflect the higher activity rates of the translation and ribosomal machinery required for the efficient translation of plastid genes during glyphosate resistance. We also observed down-regulation of *atp*F, which is an H^+^ATPase (CF_0_ subunit of the CF_0_CF_1_) and a critical component for energy production also associated with the down-regulation of *ndh*A and *ndh*B. Members of the NADH-like dehydrogenases play an important role in the PSI cyclic electron flow (Suorsa et al., 2009). Overall, these findings suggest that glyphosate has immediate effects on the photo-accumulation events and thus alters the transport and the energy production in plastid genes. Similar disruptions were reported in mitochondrial ATP influx-outflux during glyphosate resistance (Gomes et al., 2017).

## Conclusion

The inclusion of historical museum specimens in phylogenomic analyses of biodiversity provides new possibilities for both fundamental and applied plant biology research. Our study is a good example of how herbarium specimens can be used to investigate phylogeny and genomic patterns of herbicide resistance. However, it should be kept in mind that museomics are limited by the amount of plant material, and disruptive sampling should be cautiously carried out with such specimens. Herbarium genomics of weeds could also improve our understanding of resistance to herbicides. As demonstrated in our study, plastid genomes reconstructed from herbarium specimens, coupled with transcriptomic resources can also be used to investigate herbicide resistance with potential for further applications in weed management.

## Author Contributions

PP conceived the study, performed the sampling, laboratory experiments, genome assembly, annotation, and data curation, and prepared the original draft of the manuscript. GS conceived the methodologies and performed the further analyses. JH and AA performed parsimony and inverted repeat analyses, respectively. JH, XH, and PP carried out the project administration and provided resources for the work. PP and JH supervised the work. GS, AA, and PP performed the validation and visualization of the work. All authors reviewed and edited the manuscript.

## Conflict of Interest Statement

The authors declare that the research was conducted in the absence of any commercial or financial relationships that could be construed as a potential conflict of interest.

## Supplementary Material

FIGURE S1 LASTZ dot-plot comparison among Asteraceae complete plastid genome sequences.

FIGURE S2 Inverted repeat plot of 30 Asteraceae chloroplast genomes.

FIGURE S3 Phylogenetic tree of Asteraceae based on the analysis of fifty protein coding genes with parsimony used as an optimality criterion.

FIGURE S4 Evaluation of the skewness and compositional heterogeneity across the combined and partition-specific variations of the long genes *ndh*D, *ndh*H, *psa*A, *psa*B, *psb*A, *psb*B, *psb*C, and *rbc*L.

TABLE S1 List of the sequenced plastomes used for the phylogenomic analysis.

TABLE S2 Summary of the aligned transcriptomics reads.

TABLE S3 List of genes in the chloroplast genome of *Ambrosia trifida.*

TABLE S4 Genes having an intron in the *Ambrosia trifida* plastid genome, and the length of the exons and introns.

TABLE S5 Intronic reads per kb per million mapped (RPKM) across the herbicide treatments in the plastid genome of giant ragweed (*Ambrosia trifida*).
