# Supplementary figures and images for: Sequencing the Plastid Genome of Giant Ragweed (Ambrosia trifida, Asteraceae) From a Herbarium Specimen
Source: Front Plant Sci. 2019 Feb 28;10:218. doi: 10.3389/fpls.2019.00218 (PMC6403193; doi:10.3389/fpls.2019.00218)

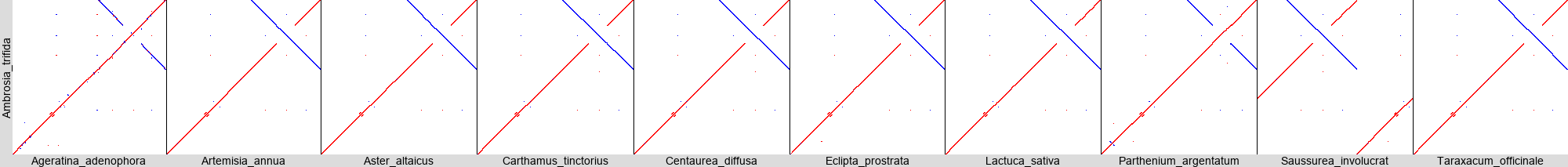

Supplement: FIGURE S1 — LASTZ dot-plot comparison among Asteraceae complete plastid genome sequences. [file Image_1.tiff]

# Inverted Repeat plot of 30 chloroplast genomes of Asteraceae

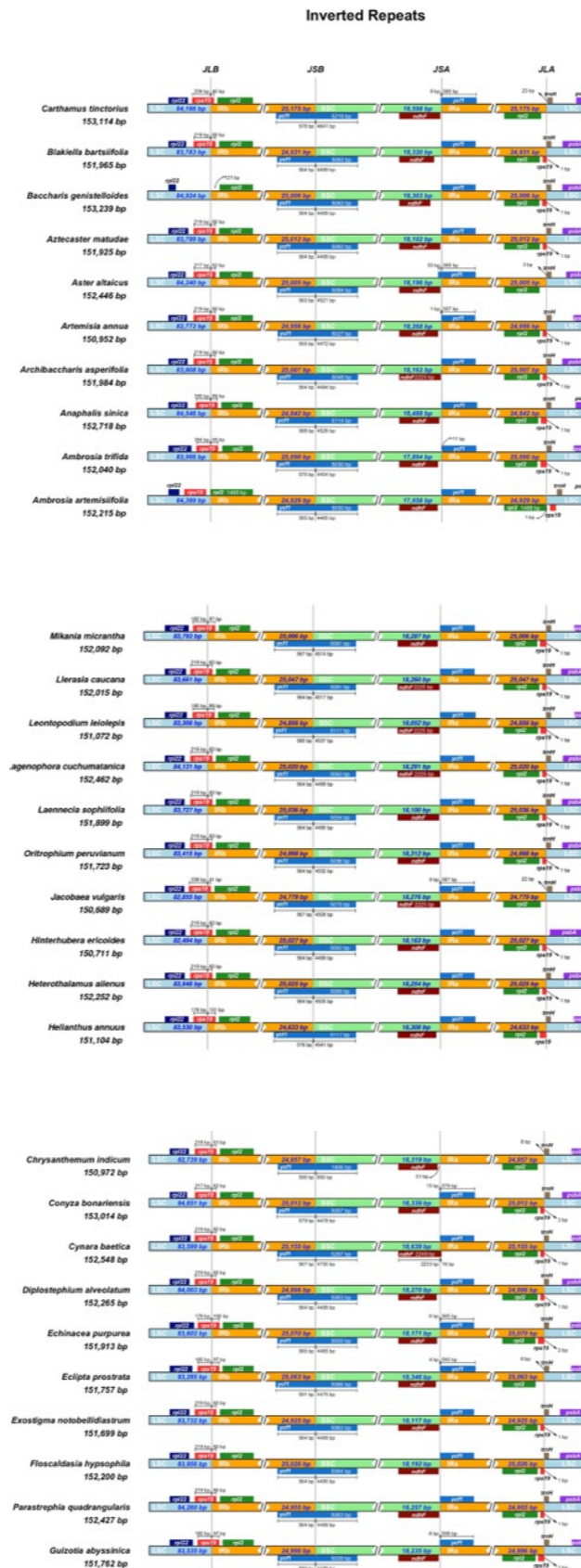

Supplement: FIGURE S2 — Inverted repeat plot of 30 Asteraceae chloroplast genomes. [file Image_2.pdf]

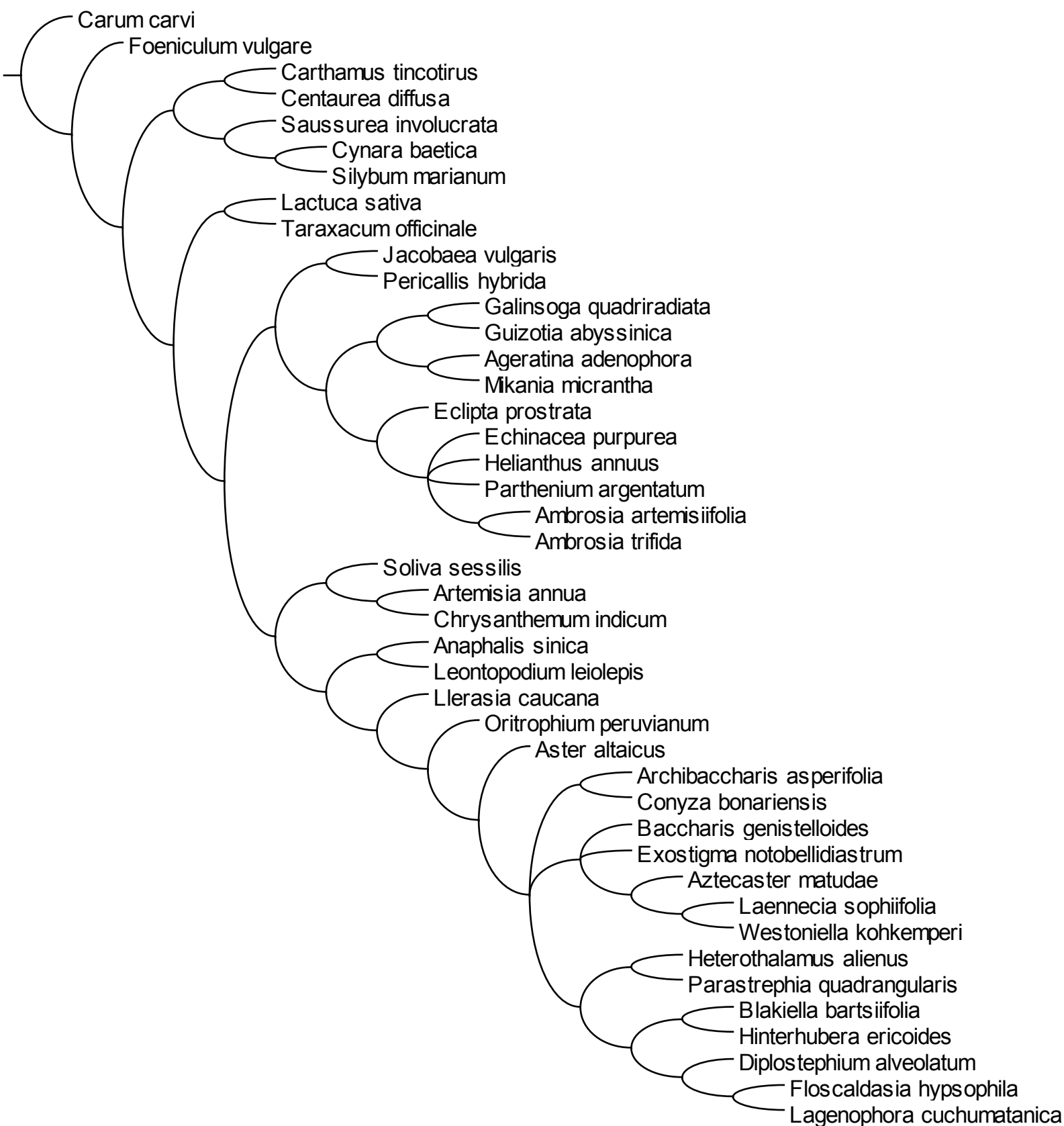

consensus of 6 EPT's of the length 7297 steps, CI 0.65 RI 0.83

Supplement: FIGURE S3 — Phylogenetic tree of Asteraceae based on the analysis of fifty protein coding genes with parsimony used as an optimality criterion. [file Image_3.pdf]

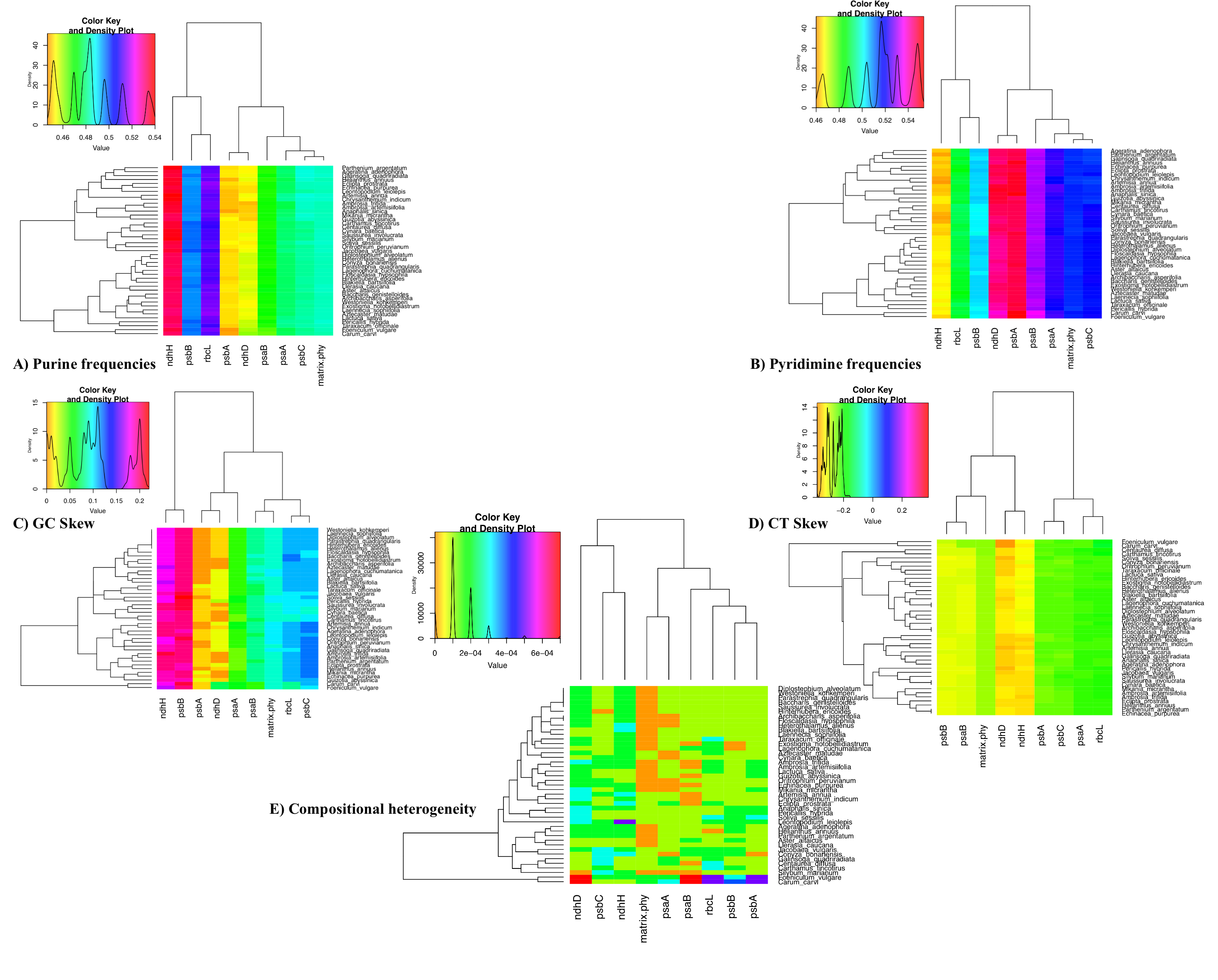

Supplement: FIGURE S4 — Evaluation of the skewness and compositional heterogeneity across the combined and partition-specific variations of the long genes ndhD, ndhH, psaA, psaB, psbA, psbB, psbC, and rbcL. [file Image_4.tiff]
