# Supplementary material for: Sequencing the Plastid Genome of Giant Ragweed (Ambrosia trifida, Asteraceae) From a Herbarium Specimen
Source: Front Plant Sci. 2019 Feb 28;10:218. doi: 10.3389/fpls.2019.00218 (PMC6403193; doi:10.3389/fpls.2019.00218)
Supplement: TABLE S1 — List of the sequenced plastomes used for the phylogenomic analysis. [file Table_1.docx]

**Supplementary Table 1: List of the sequenced plastomes used for the phylogenomic analysis**

| #Organism/Name | RefSeq |
| --- | --- |
| *Ageratina adenophora* | NC_015621.1 |
| *Ambrosia artemisiifolia* | NC_035875.1 |
| *Anaphalis sinica* | NC_034648.1 |
| *Archibaccharis asperifolia* | NC_034848.1 |
| *Artemisia annua* | NC_034683.1 |
| *Aster altaicus* | NC_034996.1 |
| *Aztecaster matudae* | NC_034898.1 |
| *Baccharis genistelloides* | NC_034852.1 |
| *Blakiella bartsiifolia* | NC_034866.1 |
| *Carthamus tinctorius* | NC_030783.1 |
| *Centaurea diffusa* | NC_024286.1 |
| *Chrysanthemum indicum* | NC_020320.1 |
| *Conyza bonariensis* | NC_035884.1 |
| *Cynara baetica* | NC_028005.1 |
| *Diplostephium alveolatum* | NC_034847.1 |
| *Echinacea purpurea* | NC_034327.1 |
| *Eclipta prostrata* | NC_030773.1 |
| *Exostigma notobellidiastrum* | NC_034864.1 |
| *Floscaldasia hypsophila* | NC_034888.1 |
| *Galinsoga quadriradiata* | NC_031853.1 |
| *Guizotia abyssinica* | NC_010601.1 |
| *Helianthus annuus* | NC_007977.1 |
| *Heterothalamus alienus* | NC_034855.1 |
| *Hinterhubera ericoides* | NC_034884.1 |
| *Jacobaea vulgaris* | NC_015543.1 |
| *Lactuca sativa* | NC_007578.1 |
| *Laennecia sophiifolia* | NC_034877.1 |
| *Lagenophora cuchumatanica* | NC_034819.1 |
| *Leontopodium leiolepis* | NC_027835.1 |
| *Llerasia caucana* | NC_034821.1 |
| *Mikania micrantha* | NC_031833.1 |
| *Oritrophium peruvianum* | NC_034849.1 |
| *Parastrephia quadrangularis* | NC_034890.1 |
| *Parthenium argentatum* | NC_013553.1 |
| *Pericallis hybrida* | NC_031898.1 |
| *Saussurea involucrata* | NC_029465.1 |
| *Silybum marianum* | NC_028027.1 |
| *Soliva sessilis* | NC_034851.1 |
| *Taraxacum officinale* | NC_030772.1 |
| *Westoniella kohkemperi* | NC_034889.1 |
| *Foeniculum vulgare* | NC_029469.1 |
| *Carum carvi* | NC_029889.1 |
