# Supplementary material for: Sequencing the Plastid Genome of Giant Ragweed (Ambrosia trifida, Asteraceae) From a Herbarium Specimen
Source: Front Plant Sci. 2019 Feb 28;10:218. doi: 10.3389/fpls.2019.00218 (PMC6403193; doi:10.3389/fpls.2019.00218)
Supplement: TABLE S2 — Summary of the aligned transcriptomics reads. [file Table_2.docx]

**Supplementary Table 2: Summary of the aligned transcriptomic reads.**

| Species | SRA Accession | Counts |
| --- | --- | --- |
|  | SRR1661420 | 227010 |
|  | SRR1661463 | 1338689 |
|  | SRR1661464 | 1168067 |
| *Ambrosia trifida* | SRR1661465 | 2310060 |
|  | SRR1661466 | 378768 |
|  | SRR1661467 | 718959 |
|  | SRR1661468 | 2790511 |
|  | SRR1661469 | 616933 |
|  |  |  |
