# Supplementary material for: Sequencing the Plastid Genome of Giant Ragweed (Ambrosia trifida, Asteraceae) From a Herbarium Specimen
Source: Front Plant Sci. 2019 Feb 28;10:218. doi: 10.3389/fpls.2019.00218 (PMC6403193; doi:10.3389/fpls.2019.00218)
Supplement: TABLE S3 — List of genes in the chloroplast genome of Ambrosia trifida. [file Table_3.docx]

**Supplementary Table 3.** – List of genes in the chloroplast genome of *A. trifida*

|  | Group of genes | Name of genes |
| --- | --- | --- |
| Protein synthesis and DNA-replication | Transfer RNAs | *trn*A(UGC)^b^, C(GCA), D(GUC), E(UUC), fM(CAU), F(GAA), G(GCC)^b^, G(UCC), I(CAU), I(GAU)^b^, K(UUU)a, L(UAA)^b^, L(UAG), L(CAA), M(CAU), N(GUU), P(UGG), Q(CUG), R(ACG), R(UCU), S(GGA), S(UGA), T(GGU), T(UGU), V(UAC)^b^, V(GAC), W(CCA), Y(GUA) |
|  | Ribosomal RNAs | *rrn*23, 16, 5, 4.5 |
|  | Ribosomal protein small subunit | *rps*2, 3, 4, 7, 8, 11, 12^b, c^, 14, 15, 16^b^, 18, 19 |
|  | Ribosomal protein large subunit | *rpl*2^b^, 14, 16^b^, 20, 22, 23, 32, 33, 36 |
|  | Subunits of of RNA polymerase | *rpo*A, C1^b^, C2 |
| Photosynthesis | Photosystem I | *psa*A, B, C, I, J |
|  | Photosystem II | *psb*A, B, C, D, E, F, H, I, J, K, L, M, N, T, Z |
|  | Cytochrome b/f complex | *pet*A, B^b^, D^b^, G, L, N |
|  | ATP synthase | *atp*A, B, E, F^b^, H, I |
|  | NADH-dehydrogenase | *ndh*A^b^, B^b^, C, D, E, F, G, H, I, J, K |
|  | Large subunit Rubisco | *rbc*L |
| Miscellaneous group | Translation initiation factor IF-1 | infA^d^ |
|  | Acetyl-CoA carboxylase | *acc*D |
|  | Cytochrome c biogenesis | *ccs*A |
|  | Maturase | *mat*K |
|  | ATP-dependent protease | *clp*P^a^ |
|  | Inner membrane protein | *cem*A |
|  |  |  |
|  | Assembly of the photosystem I | *ycf*3^a^, *ycf*4 |
| Unknown function | Conserved hypothetical chloroplast ORF | *ycf*1^d,*^, *ycf*2, *ycf*15^d,*^ |

^a^Gene containing two introns; ^b^Gene containing a single intron; ^c^Gene divided into two independent transcription units; ^d^Pseudogene
