# Supplementary material for: Sequencing the Plastid Genome of Giant Ragweed (Ambrosia trifida, Asteraceae) From a Herbarium Specimen
Source: Front Plant Sci. 2019 Feb 28;10:218. doi: 10.3389/fpls.2019.00218 (PMC6403193; doi:10.3389/fpls.2019.00218)
Supplement: TABLE S4 — Genes having an intron in the Ambrosia trifida plastid genome, and the length of the exons and introns. [file Table_4.docx]

**Supplementary Table 4.** The genes having intron in the *Ambrosia trifida* plastid genome and the length of the exons and introns.

| **Gene** | **Location** | **Exon1 (bp)** | **Intron1 (bp)** | **Exon2 (bp)** | **Intron2 (bp)** | **Exon3 (bp)** |
| --- | --- | --- | --- | --- | --- | --- |
| *rps*16 | LSC | 41 | 853 | 224 |  |  |
| *trn*G-GCC | LSC | 46 | 753 | 22 |  |  |
| *atp*F | LSC | 144 | 708 | 409 |  |  |
| *rpo*C1 | LSC | 452 | 770 | 1637 |  |  |
| *ycf*3 | LSC | 131 | 694 | 227 | 739 | 152 |
| *trn*L-UAA^a^ | LSC | 34 | 437 | 48 |  |  |
| *trn*V-UAC | LSC | 34 | 579 | 44 |  |  |
| *rps*12^b^ | LSC | 107 |  | 245 | 521 | 25 |
| *clp*P | LSC | 70 | 751 | 291 | 633 | 227 |
| *pet*B | LSC | 5 | 757 | 641 |  |  |
| *pet*D | LSC | 6 | 716 | 475 |  |  |
| *rpl*16 | LSC | 8 | 1016 | 398 |  |  |
| *rpl*2 | IR | 392 | 662 | 434 |  |  |
| *ndh*B | IR | 682 | 671 | 755 |  |  |
| *trn*I-GAU | IR | 34 | 775 | 41 |  |  |
| *trn*A-UGC | IR | 34 | 815 | 37 |  |  |
| *ndh*A | SSC | 552 | 1054 | 538 |  |  |

^a^*trn*L-UAA contains the only group I intron, others belong to group II

^b^*rps*12 is a trans-spliced gene with 5' end exon located in the LSC region and the duplicated 3'end exon located in the IR regions.
