# Supplementary material for: Sequencing the Plastid Genome of Giant Ragweed (Ambrosia trifida, Asteraceae) From a Herbarium Specimen
Source: Front Plant Sci. 2019 Feb 28;10:218. doi: 10.3389/fpls.2019.00218 (PMC6403193; doi:10.3389/fpls.2019.00218)
Supplement: TABLE S5 — Intronic reads per kb per million mapped (RPKM) across the herbicide treatments in the plastid genome of giant ragweed (Ambrosia trifida). [file Table_5.docx]

| START | END | RPKM_0 | RPKM_3 | RPKM_8 | RPKM_12 |
| --- | --- | --- | --- | --- | --- |
| 1767 | 4331 | 364.227599 | 54.7721935 | 104.84407 | 104.84407 |
| 5456 | 6307 | 833.395838 | 217.691978 | 249.490703 | 249.490703 |
| 16758 | 17526 | 206.488701 | 19.4531117 | 31.212536 | 31.212536 |
| 27264 | 27970 | 249.5803 | 84.6458345 | 87.3092064 | 87.3092064 |
| 30418 | 31169 | 392.997581 | 59.6803853 | 160.735358 | 160.735358 |
| 42555 | 43292 | 651.499402 | 164.197988 | 518.083327 | 518.083327 |
| 43521 | 44213 | 38.1944418 | 15.1127064 | 24.743216 | 24.743216 |
| 47331 | 47766 | 151.899734 | 75.5585691 | 90.5317301 | 90.5317301 |
| 51169 | 51746 | 458.068522 | 167.006818 | 298.230797 | 298.230797 |
| 70057 | 70688 | 32238.8529 | 5554.55088 | 9737.46327 | 9737.46327 |
| 70981 | 71310 | 15906.5338 | 1877.71604 | 4639.67639 | 4639.67639 |
| 74724 | 75479 | 1295.27217 | 288.905763 | 317.499704 | 317.499704 |
| 76323 | 77039 | 1784.18542 | 338.027702 | 473.493923 | 473.493923 |
| 81428 | 82442 | 234.590713 | 33.8875508 | 70.9207919 | 70.9207919 |
| 84559 | 85219 | 1321.52769 | 306.722518 | 537.017763 | 537.017763 |
| 94889 | 95558 | 375.321764 | 92.6770667 | 116.452152 | 116.452152 |
| 97173 | 97707 | 10146.5609 | 1335.92605 | 2024.85691 | 2024.85691 |
| 101984 | 102762 | 1245.65592 | 497.359557 | 631.632606 | 631.632606 |
| 102900 | 103713 | 1273.30466 | 497.080841 | 612.864809 | 612.864809 |
| 116003 | 117055 | 435.484409 | 130.653903 | 133.462837 | 133.462837 |
| 132294 | 133107 | 1376.25269 | 502.593753 | 627.607261 | 627.607261 |
| 133245 | 134018 | 1219.52102 | 506.374816 | 636.825721 | 636.825721 |
| 140449 | 141118 | 348.983395 | 90.4438844 | 106.214601 | 106.214601 |
| 150788 | 151448 | 1294.83016 | 302.195248 | 524.046319 | 524.046319 |

**Supplementary Table 5**. a) Intronic reads per kilo base per million mapped (RPKM) across the sensitive treatments in the plastid genome of giant ragweed (*Ambrosia trifida*).

**Supplementary Table 5:** b) Intronic reads per kilo base per million mapped (RPKM) across the resistant treatments in the plastid genome of giant ragweed (*Ambrosia trifida*).

| START | END | RPKM_0 | RPKM_3 | RPKM_8 | RPKM_12 |
| --- | --- | --- | --- | --- | --- |
| 1767 | 4331 | 27.1821995 | 134.890074 | 220.786372 | 103.705585 |
| 5456 | 6307 | 52.3943437 | 291.625175 | 310.541693 | 106.117557 |
| 16758 | 17526 | 15.7824183 | 123.756495 | 159.373947 | 106.387325 |
| 27264 | 27970 | 31.8841896 | 190.718224 | 457.066256 | 123.851533 |
| 30418 | 31169 | 38.0435239 | 158.197383 | 211.135271 | 177.031371 |
| 42555 | 43292 | 74.0081924 | 293.746759 | 418.96849 | 164.834635 |
| 43521 | 44213 | 6.88118653 | 11.4456874 | 52.2592447 | 47.1250213 |
| 47331 | 47766 | 55.7282634 | 48.5542723 | 76.7393047 | 183.709607 |
| 51169 | 51746 | 78.775329 | 228.781504 | 371.228049 | 385.063308 |
| 70057 | 70688 | 2257.06091 | 11895.2677 | 14305.7692 | 2945.23135 |
| 70981 | 71310 | 888.146512 | 9364.86837 | 12513.8714 | 2309.17169 |
| 74724 | 75479 | 177.742581 | 685.386962 | 1536.43777 | 835.375968 |
| 76323 | 77039 | 180.168964 | 634.223228 | 1363.70353 | 574.572885 |
| 81428 | 82442 | 11.0997228 | 187.465459 | 373.101353 | 86.2319352 |
| 84559 | 85219 | 84.6101597 | 532.027921 | 676.483132 | 212.84252 |
| 94889 | 95558 | 38.1770735 | 244.676518 | 288.99116 | 81.9561548 |
| 97173 | 97707 | 336.421373 | 1443.67127 | 1706.06635 | 635.513796 |
| 101984 | 102762 | 224.790787 | 1242.01891 | 1669.79499 | 2364.32911 |
| 102900 | 103713 | 150.153446 | 948.241646 | 1493.54934 | 1666.6039 |
| 116003 | 117055 | 43.6181122 | 230.886642 | 499.772017 | 130.807229 |
| 132294 | 133107 | 152.815741 | 1055.40594 | 1620.15031 | 1755.64224 |
| 133245 | 134018 | 231.844921 | 1243.22178 | 1655.40479 | 2364.32374 |
| 140449 | 141118 | 39.4712116 | 236.783727 | 280.674867 | 81.9561548 |
| 150788 | 151448 | 83.2983743 | 484.025402 | 680.697981 | 210.670658 |
