# Supplementary material for: Sequencing the Plastid Genome of Giant Ragweed (Ambrosia trifida, Asteraceae) From a Herbarium Specimen
Source: Front Plant Sci. 2019 Feb 28;10:218. doi: 10.3389/fpls.2019.00218 (PMC6403193; doi:10.3389/fpls.2019.00218)
Supplement: Supplementary file 10 [file Data_Sheet_1.docx]

**Supplementary Data 1** - Defining most recent common ancestors (mrca) for PATHd8.

mrca:Foeniculum_vulgare,Ambrosia_trifida,fixage=92;mrca:Lactuca_sativa,Helianthus_annuus,minage=39;mrca:Soliva_sessilis,Artemisia_annua,minage=23;mrca:Carthamus_tincotirus,Helianthus_annuus,minage=38;mrca:Carthamus_tincotirus,Lactuca_sativa,minage=33;mrca:Mikania_micrantha,Ambrosia_trifida, minage=24
